# Supplementary material for: Selecting the Most Relevant Brain Regions to Classify Children with Developmental Dyslexia and Typical Readers by Using Complex Magnocellular Stimuli and Multiple Kernel Learning
Source: Brain Sci. 2021 May 28;11(6):722. doi: 10.3390/brainsci11060722 (PMC8228080; doi:10.3390/brainsci11060722)
Supplement: Supplementary file 1 [file brainsci-11-00722-s001.zip › Supplementary Files/Table S1.pdf]

Table S1a.
Bivariate correlations within reading in the total sample (n=44).

	TR, speed	SWR, accuracy	SWR, speed	SPWR, accuracy	SPWR, speed
**TR, accuracy**	0.852**	0.731**	0.823**	0.853**	0.716**
**TR, speed**	1	0.670**	0.780**	0.732**	0.735**
**SWR, accuracy**		1	0.777**	0.709**	0.650**
**SWR, speed**			1	0.867**	0.875**
**SPWR, accuracy**				1	0.767**

TR=Text reading; SWR=single words reading; SPWR=single pseudo-words reading.
**Two-tailed p-value < 0.01

Table S1b.
Bivariate correlations within VWM in the total sample (n=44).

	SLBS	SDFS	SDBS
**SLFS**	0.292	0.426**	0.462**
**SLBS**	1	0.358*	0.540**
**SDFS**		1	0.268

SLFS=single letters forward span; SLBS=single letters backward span; SDFS=single digits forward span; SDBS=single digits backward span; SNWR=single non-word repetition.
**Two-tailed p-value < 0.01; * two-tailed p-value < 0.05
