# Supplementary material for: Selecting the Most Relevant Brain Regions to Classify Children with Developmental Dyslexia and Typical Readers by Using Complex Magnocellular Stimuli and Multiple Kernel Learning
Source: Brain Sci. 2021 May 28;11(6):722. doi: 10.3390/brainsci11060722 (PMC8228080; doi:10.3390/brainsci11060722)
Supplement: Supplementary file 1 [file brainsci-11-00722-s001.zip › Supplementary Files/Table S2.pdf]

Table S2. Correlations between the mean activation of each contrast map within each significant ROIs and the neuropsychological domains in the total sample (n=44).

	M-*vs*-B	P-*vs*-B	CML6-*vs*-B	CML15-*vs*-B	CML40-*vs*-B
	r (*p*)	*r* (*p*)	*r* (*p*)	*r* (*p*)	*r* (*p*)
	**Right Lateral Area 7P**				
**Reading†**	*0.312 (0.039)*	0.211 (0.168)	*0.552 (<0.001)**	*0.409 (0.006)*	*0.388 (0.009)*
**VWM‡**	0.203 (0.191)	0.149 (0.339)	0.271 (0.079)	0.294 (0.056)	0.192 (0.218)
**SNWR§**	*0.323 (0.033)*	*0.309 (0.042)*	0.272 (0.074)	*0.324 (0.032)*	0.259 (0.090)
	**Left Area PGp**				
**Reading†**	0.249 (0.102)	0.167 (0.277)	*0.376 (0.012)*	*0.348 (0.021)*	0.223 (0.146)
**VWM‡**	-0.013 (0.932)	-0.030 (0.851)	0.171 (0.273)	0.131 (0.402)	0.094 (0.548)
**SNWR§**	0.083 (0.593)	0.091 (0.558)	0.183 (0.235)	0.212 (0.167)	0.066 (0.672)
	**Left Area V6A**				
**Reading†**	0.132 (0.392)	-0.076 (0.624)	*0.311 (0.040)*	0.259 (0.090)	0.083 (0.590)
**VWM‡**	0.040 (0.801)	-0.047 (0.765)	0.117 (0.453)	0.055 (0.728)	-0.061 (0.699)
**SNWR§**	0.168 (0.275)	0.081 (0.601)	0.205 (0.182)	*0.305 (0.044)*	0.172 (0.263)
	**Right Medial Area 7A**				
**Reading†**	0.192 (0.212)	0.170 (0.269)	*0.540 (<0.001)**	0.256 (0.093)	0.297 (0.050)
**VWM‡**	0.159 (0.308)	0.102 (0.516)	*0.321 (0.036)*	0.267 (0.084)	0.210 (0.176)
**SNWR§**	0.237 (0.122)	0.188 (0.222)	*0.322 (0.033)*	*0.350 (0.020)*	*0.322 (0.033)*
	**Left Ventro-Medial Visual Area 1**				
**Reading†**	-0.032 (0.837)	-0.236 (0.122)	0.263 (0.085)	0.280 (0.065)	0.210 (0.170)
**VWM‡**	0.111 (0.477)	0.045 (0.773)	*0.302 (0.049)*	0.262 (0.089)	0.285 (0.064)
**SNWR§**	-0.080 (0.605)	0.083 (0.593)	*0.419 (0.005)*	*0.477 (0.001)*	*0.360 (0.016)*
	**Left Area Lateral Occipital 2**				
**Reading†**	0.252 (0.099)	-0.121 (0.433)	0.286 (0.059)	0.260 (0.088)	*0.319 (0.035)*
**VWM‡**	*0.419 (0.005)*	0.251 (0.104)	*0.442 (0.003)*	*0.315 (0.039)*	*0.410 (0.006)*
**SNWR§**	*0.307 (0.043)*	0.115 (0.458)	0.231 (0.132)	*0.473 (0.001)*	*0.329 (0.029)*
	**Right Area IFJ posterior**				
**Reading†**	*0.461 (0.002)*	0.208 (0.175)	*0.306 (0.043)*	0.123 (0.428)	0.167 (0.279)
**VWM‡**	0.227 (0.144)	0.083 (0.598)	0.083 (0.595)	0.050 (0.749)	0.076 (0.626)
**SNWR§**	0.180 (0.242)	0.178 (0.247)	0.220 (0.151)	0.023 (0.880)	-0.034 (0.827)
	**Right Ventro-Medial Visual Area 1**				
**Reading†**	*0.331 (0.028)*	0.143 (0.354)	0.198 (0.197)	0.206 (0.179)	0.177 (0.251)
**VWM‡**	0.215 (0.166)	0.212 (0.173)	0.127 (0.417)	-0.005 (0.976)	0.044 (0.777)
**SNWR§**	0.170 (0.270)	0.158 (0.305)	*0.370 (0.014)*	*0.393 (0.008)*	*0.303 (0.046)*
	**Right Area 5-L**				

**Reading**†	0.004 (0.978)	0.000 (0.999)	*0.492 (0.001)*	0.257 (0.092)	0.191 (0.214)
**VWM**‡	-0.059 (0.708)	-0.066 (0.675)	0.279 (0.071)	0.300 (0.050)	0.201 (0.197)
**SNWR**§	-0.118 (0.446)	-0.118 (0.447)	*0.322 (0.033)*	*0.414 (0.005)*	*0.321 (0.034)*
	**Right Area PH**				
**Reading**†	*0.428 (0.004)*	*0.314 (0.038)*	0.215 (0.162)	0.221 (0.150)	0.176 (0.254)
**VWM**‡	0.273 (0.077)	0.161 (0.304)	-0.031 (0.843)	0.035 (0.823)	-0.022 (0.891)
**SNWR**§	*0.383 (0.010)*	*0.337 (0.025)*	*0.391 (0.009)*	*0.420 (0.005)*	*0.324 (0.032)*
	**Right Ventro-Medial Visual Area 2**				
**Reading**†	0.289 (0.057)	0.070 (0.652)	0.173 (0.263)	0.179 (0.246)	0.121 (0.434)
**VWM**‡	0.268 (0.083)	0.116 (0.459)	0.136 (0.385)	0.011 (0.945)	0.058 (0.713)
**SNWR**§	0.123 (0.428)	0.161 (0.297)	*0.332 (0.028)*	0.273 (0.073)	0.194 (0.206)

Uncorrected significant correlations are reported in italics.
* Significant after Bonferroni correction for multiple comparisons.
M-*vs*-B=M stimulus *versus* Baseline; P-*vs*-B=M stimulus *versus* Baseline; CML6-*vs*-B=Coherent Motion Level 6% *versus* Baseline; CML15-*vs*-B=Coherent Motion Level 15% *versus* Baseline; CML40-*vs*-B=Coherent Motion Level 40% *versus* Baseline; SNWR=single non-words repetition.

† Mean score of text [109-110], single unrelated words and pronounceable pseudo-words [111-112] reading tests.

‡ Mean score of the Single Digit Forward Span, Single Digit Backward Span, Single Letter Forward Span, Single Letter Backward Span tasks [113].

§ As assessed by the non-word repetition test [114].
