# Supplementary figures and images for: Selecting the Most Relevant Brain Regions to Classify Children with Developmental Dyslexia and Typical Readers by Using Complex Magnocellular Stimuli and Multiple Kernel Learning
Source: Brain Sci. 2021 May 28;11(6):722. doi: 10.3390/brainsci11060722 (PMC8228080; doi:10.3390/brainsci11060722)

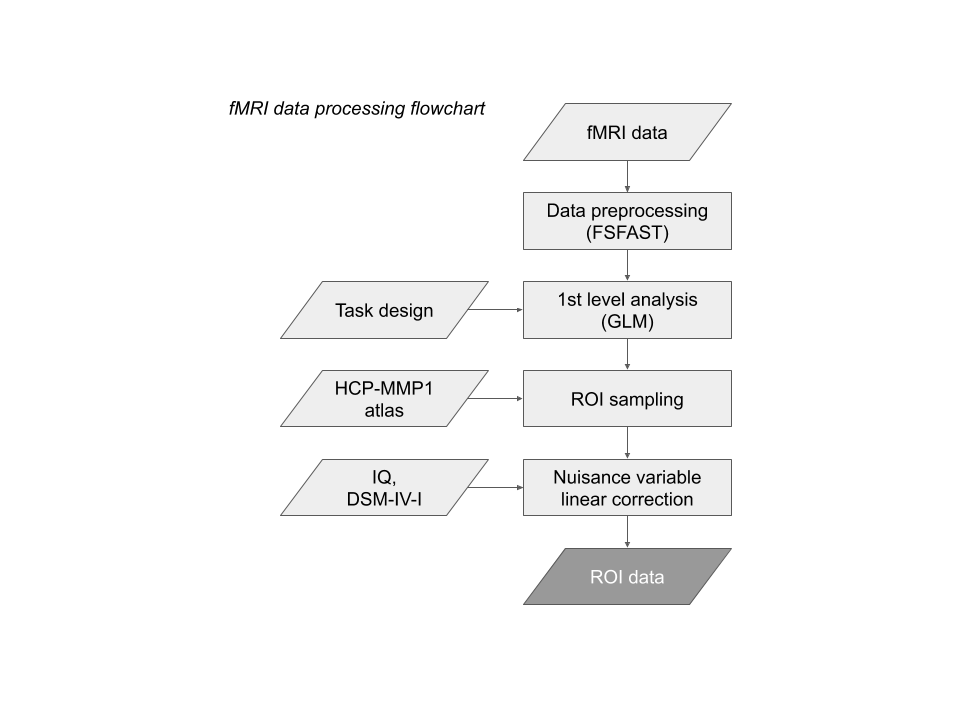

Supplement: Supplementary file 1 [file brainsci-11-00722-s001.zip › Supplementary Files/Supplementary Figure S1.png]

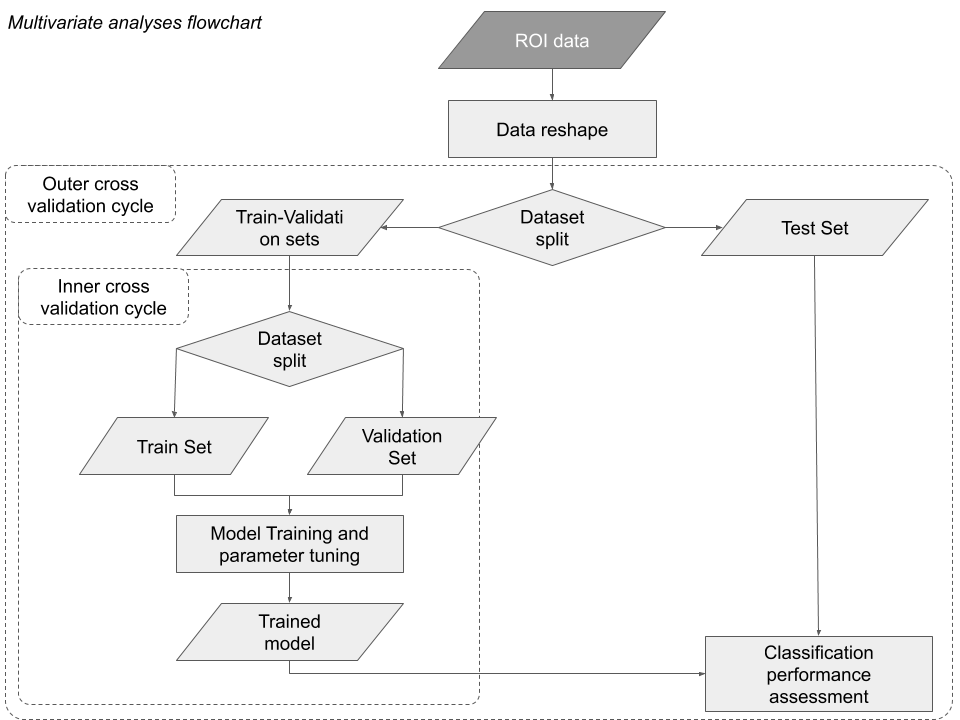

Supplement: Supplementary file 1 [file brainsci-11-00722-s001.zip › Supplementary Files/Supplementary Figure S2.png]
